# Learning from the COVID-19 pandemic to strengthen routine immunization systems

**DOI:** 10.1371/journal.pmed.1003934

**Published:** 2022-02-22

**Authors:** Kate Causey, Jonathan F. Mosser

**Affiliations:** 1 Institute for Health Metrics and Evaluation, University of Washington, Seattle, Washington, United States of America; 2 Department of Health Metrics Sciences, School of Medicine, University of Washington, Seattle, Washington, United States of America; 3 Pediatric Infectious Diseases, Seattle Children’s Hospital, Seattle, Washington, United States of America

## Abstract

Kate Causey and Jonathan F Mosser discuss what can be learnt from the observed impacts of the COVID-19 pandemic on routine immunisation systems.

In the final months of 2021, deaths due to the Coronavirus Disease 2019 (COVID-19) surpassed 5 million globally [1]. Available data suggest that even this staggering figure may be a substantial underestimate of the true toll of the pandemic [2]. Beyond mortality, it may take years to fully quantify the direct and indirect impacts of the COVID-19 pandemic such as disruptions in preventive care services. In an accompanying research study in *PLOS Medicine*, McQuaid and colleagues report on the uptake of routine childhood immunizations in 2020 in Scotland and England during major pandemic-related lockdowns [3]. This adds to a growing body of literature quantifying the impact of the COVID-19 pandemic on routine health services and childhood immunization [4,5], which provides important opportunities to learn from early pandemic experiences as immunization systems face ongoing challenges.

McQuaid and colleagues compared weekly or monthly data on vaccine uptake in Scotland and England from January to December of 2020 to the rates observed in 2019 to estimate the changes in uptake before, during, and after COVID-19 pandemic lockdowns in each country. The authors included 2 different preschool immunizations, each with multiple doses. They found significantly increased uptake within 4 weeks of eligibility during the lockdown and postlockdown periods in Scotland for all 5 vaccine dose combinations examined: During lockdown, percentage point increases ranged from 1.3% to 14.3%. In England, there were significant declines in uptake during the prelockdown, lockdown, and postlockdown periods for all 4 vaccine dose combinations examined. However, declines during lockdown were small, with percentage point decreases ranging from −0.5% to −2.1%. Due to the nature of the data available, the authors were unable to account for possible seasonal variation in vaccine delivery, control for important individual-level confounders or effect modifiers such as child sex and parental educational attainment, or directly compare outcomes across the 2 countries.

These findings stand in contrast to the documented experience of many other countries, where available data suggest historic disruptions in routine childhood vaccination coverage, particularly during the first months of pandemic-related lockdowns [5,6]. Supply side limitations such as delayed shipments of vaccines and supplies [7], inadequate personal protective equipment [8], staff shortages [9], and delayed or canceled campaigns and introductions [9] threatened vaccine delivery. Furthermore, fear of exposure to COVID-19 at vaccination centers [10], misinformation about vaccine safety [8], and lockdown-related limitations on travel to facilities [9,10] reduced demand. In polls of country experts conducted by WHO, UNICEF, and Gavi, the Vaccine Alliance throughout the second quarter of 2020, 126 of 170 countries reported at least some disruption to routine immunization programs [10,11]. Global estimates suggest that millions more children missed doses of important vaccines than would have in the absence of the COVID-19 pandemic [5,6]. While many vaccine programs showed remarkable resilience in the second half of 2020, with rates of vaccination returning to or even exceeding prepandemic levels [5,6], disruptions to immunization services persisted into 2021 in many countries [12].

As the authors discuss, it is critical to pinpoint the specific program policies and strategies that contributed to increased uptake in Scotland and only small declines in England and, more broadly, to the rapid recovery of vaccination rates observed in many other countries. McQuaid and colleagues cite work suggesting that increased flexibility in parental working patterns during lockdowns, providing mobile services or public transport to vaccine centers, and sending phone- and mail-based reminders are strategies that may have improved uptake of timely vaccination in Scotland during this period [13]. Similarly, immunization programs around the world have employed a broad range of strategies to maintain or increase vaccination during the pandemic. Leaders in Senegal, Paraguay, and Sri Lanka designed and conducted media campaigns to emphasize the importance of childhood immunization even during lockdown [8,14,15]. Although many programs delayed mass campaigns in the spring of 2020, multiple countries were able to implement campaigns by the summer of 2020 [8,16–20]. In each of these examples, leaders responded quickly to meet the unique challenges presented by the COVID-19 pandemic in their communities.

Increased data collection and tracking systems are essential for efficient and effective responses as delivery programs face challenges. When concern arose for pandemic-related disruptions to immunization services, public health decision-makers in Scotland and England responded by increasing the frequency and level of detail in reports of vaccine uptake and by making these data available for planning and research. The potential for robust data systems to inform real-time decision-making is not limited to high-income countries. For instance, the Nigerian National Health Management Information System released an extensive online dashboard shortly after the onset of the pandemic, documenting the impact of COVID-19 on dozens of indicators of health service uptake, including 16 related to immunization [21]. Vaccination data systems that track individual children and doses, such as the reminder system in Scotland, allow for highly targeted responses. Similarly, in Senegal, Ghana, and in Karachi, Pakistan, healthcare workers have relied on existing or newly implemented tracking systems to identify children who have missed doses and provide text message and/or phone call reminders [8,22,23]. Investing in robust routine data systems allows for rapid scale-up of data collection, targeted services to those who miss doses, and a more informed response when vaccine delivery challenges arise.

Policy and program decision-makers must learn from the observed impacts of the COVID-19 pandemic on health systems and vaccine delivery. The study by McQuaid and colleagues provides further evidence that vaccination programs in England and Scotland leveraged existing strengths and identified novel strategies to mitigate disruptions and deliver vaccines in the early stages of the pandemic. However, the challenges posed by the pandemic to routine immunization services continue. To mitigate the risk of outbreaks of measles and other vaccine-preventable diseases, strategies are needed to maintain and increase coverage, while ensuring that children who missed vaccines during the pandemic are quickly caught up. The accompanying research study provides important insights into 2 countries where services were preserved—and even increased—in the early pandemic. To meet present and future challenges, we must learn from early pandemic successes such as those in Scotland and England, tailor solutions to improve vaccine uptake, and strengthen data systems to support improved decision-making.
